# Dextrose in Blood

**Published:** 1914-08

**Authors:** 


**Dextrose in Blood.** B. Purjesz, Wien. Klin. Woch., No. 26, 1913, states the normal to be 0.0451 to 0.087%, mainly in the plasma, only a trace in the erythrocytes. It is increased in fever, hypertension and following injections of infundibular pituitary extract, decreased when the thyroid is active, in Addison's disease and totally lacking in miliary tuberculosis and typhoid (differential diagnosis from pneumonia in which it is increased).

